# DNA barcoding and cryptic diversity in fishes from the Ili River Valley in China, Xinjiang

**DOI:** 10.1002/ece3.70352

**Published:** 2024-10-03

**Authors:** Ling‐Ling Zheng, Dan Yu, Ning Sun, Cheng Wang, Wen‐Jun Chen, Zu‐Fa Ding, Shun‐Ping He, Lian‐Dong Yang

**Affiliations:** ^1^ State Key Laboratory of Freshwater Ecology and Biotechnology Institute of Hydrobiology, Chinese Academy of Sciences Wuhan China; ^2^ School of Ecology and Environment Anhui Normal University Wuhu Anhui China

**Keywords:** cryptic species, cytochrome c oxidase subunit I, DNA barcoding, fish identification, genetic diversity

## Abstract

The Ili River Valley, located in the northwest of China, serves as a vital repository for fish genetic resources. Its extensive water network and diverse climate have given rise to a unique fish composition and endemic species. In this study, we collected the cytochrome c oxidase subunit I (COI) sequences from 660 fish specimens in the Ili River Valley. The effectiveness of DNA barcoding in identifying fish species in the area was assessed by examining genetic distances, constructing phylogenetic trees, and performing ABGD (Automatic Barcode Gap Discovery) analyses, among other methods. In total, 20 species were identified, including one unidentified species (*Silurus* sp.). Except for *Silurus asotus* and *Hypophthalmichthys molitrix* (only one sample), the maximum intraspecific genetic distance among the remaining species was smaller than the minimum interspecific distance, which proves that the species exhibit obvious barcode gaps. In the Neighbor‐Joining trees, 20 species formed separate monophyletic branches. According to ABGD analysis, 660 sequences were categorized into 19 Operational Taxonomic Units, with *Silurus* sp. and *S. asotus* grouped into a single OTU. The *Silurus* in this study exhibits shared haplotypes and significant genetic divergence, suggesting the potential presence of cryptic species. Furthermore, the nucleotide diversity across all species fell below the threshold level, indicating that the local fish population is gradually declining. In conclusion, this study has demonstrated the effectiveness of DNA barcoding in identifying fish species in the Ili River Valley, providing valuable data to support the conservation of local fish resources.

## Graphical abstract

With a 95% success rate in identifying species, DNA barcoding based on the mitochondrial COI gene was validated in this study using genetic diversity, species delimitation, genetic distances, and unidentified species analysis. Simultaneously, we discovered that the Ili River Valley's fish species populations are drastically decreasing to the point where the area faces a major ecological risk.

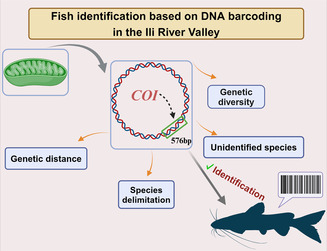

## 1 INTRODUCTION

The Ili River, an international river shared by China and Kazakhstan, flows from southeast to northwest, positioned between 74° E to 85° E longitude and 42° N to 47° N latitude (Zhu, 2021). The Ili River in Xinjiang, China, also known as the Ili River Valley, is located between the northern branch of the Tian Shan, Mount Borokonu, and the southern branch of the Tian Shan, Mount Karlik (Zhang et al., 2013). Spanning 442 kilometres in length and encompassing a drainage area of 57,600 square kilometres, the Ili River boasts the highest runoff volume in Xinjiang (Zhang, 2006). The Ili Valley is nestled among mountains on its east, north, and south sides, featuring a terrain that tapers in the east and expands in the west, creating a V‐shaped landscape that opens towards the west (Aite et al., 2018). This unique topography not only shields the valley from the dry air flow of the southern Taklimakan Desert but also welcomes warm and moist airflow from the Arctic and Atlantic Oceans, resulting in plentiful rainfall (Aite et al., 2018; Xia et al., 2018). In addition, the Ili River Valley sits at the crucial geographical juncture between Xinjiang in China and Central Asia, so the region is not only a key area for economic and trade exchanges between China and Kazakhstan, but also an important ecological barrier in the western part of China.

At the beginning of the 20th century, there were 13 indigenous fish species in the Ili River Basin, of which 10 species were distributed in the Ili River Valley in China (Ren, 1998). By the end of the 20th century, the number of fish species in the Ili River Valley had expanded to 32, belonging to 6 orders, 10 families, and 12 genera, due to the transplantation and domestication of economically valuable fish such as *Cyprinus carpio*, *Carassius auratus*, and *Hemiculter leucisculus* (Liu et al., 2022). In recent years, although the economy of the Ili Kazak Autonomous Prefecture in Xinjiang has seen rapid growth, this development has also led to certain ecological challenges. As a result of a series of human‐induced economic activities, such as hydraulic engineering, fisheries development, river pollution, and agricultural irrigation, the Ili River Valley has experienced significant wetland fragmentation and a rapid decline in rainfall, which has led to a substantial decrease in the water volume of the Ili River's main stem (Kurban et al., 2016). Currently, the ecological environment of the Ili River Valley is in a delicate state, with a marked decline in the number of fish species crucial for maintaining ecological balance (Li et al., 2017). There are currently 29 fish species in the Ili River Valley of China, almost all belonging to the Cypriniformes, with the majority being invasive species (Liu et al., 2022). Both the number of native fish species and overall fish diversity are on a steep decline. Under such a severe situation, the protection and sustainable management of fish resources in the Ili River Valley should not be delayed, and the accurate and rapid identification of fish species is the key to the protection work.

In the past, the taxonomic status of species has been largely based on morphological features. However, the identifications that rely on the subjective experience of taxonomists carry considerable risk. Consequently, a molecular approach has been created, enabling the quick and consistent identification of species using specific DNA fragments. DNA barcoding involves the use of relatively short and conserved segments of the genome that still exhibit enough variation to distinguish between species (Hebert, Cywinska, et al., 2003). The cytochrome c oxidase subunit I (COI) gene is considered to be the standard DNA barcode for most animals (Bingpeng et al., 2018; Vences et al., 2005). Compared with traditional morphological classification and identification, DNA barcoding requires only a small amount of biological tissue to identify species quickly and accurately, thus accelerating the discovery of new and cryptic species (Li et al., 2010). DNA barcoding effectively compensates for the shortcomings of traditional morphological classification methods, making it the main means of species identification (Hebert & Gregory, 2005). Furthermore, numerous studies have demonstrated the widespread application of DNA barcoding technology, with the mitochondrial COI gene as a key molecular marker in fish identification. For instance, Luo et al. applied DNA barcoding to categorize 40 fish species found off the coasts of China and Japan, identifying clear barcode gaps among these species (Luo et al., 2021). Furthermore, Ko et al., Wiwobo et al., and Hou et al. proved the suitability and applicability of DNA barcoding for juvenile fish identification (Hou et al., 2021; Ko et al., 2013; Wibowo et al., 2017). Knebelsberger et al. and Zangl et al. showed that morphologically determined species are highly congruent with DNA barcodes with respect to Central European river and lake fish species (Knebelsberger et al., 2015; Zangl et al., 2022). In brief, the emergence of DNA barcoding technology not only promotes the traditional taxonomic studies but also greatly enhances the ability of humans to monitor and protect biodiversity. Furthermore, with the continuous development and improvement of DNA barcode technology, researchers have created several key bioinformatics platforms, including the NCBI database (http://www.boldsystems.org/), BOLD database (http://www.boldsystems.org/), FISH‐BOL database (http://www.Fishbol.org/) (Zhang et al., 2022). These bioinformatics platforms have contributed to the construction of global species databases, achieving automation and standardization of species retrieval and identification.

This study uses mitochondrial COI genes as molecular markers for DNA barcodes and sequences the majority of fish species in the Ili River Valley region. We evaluate the effectiveness of DNA barcoding for fish in the Ili River Valley by analyzing genetic distances, barcode gaps, and phylogenetic trees. Nucleotide diversity and haplotype diversity of species are calculated to assess native fish biodiversity at the molecular level. In addition, we further explore the possibility of cryptic species by combining ABGD analysis (Puillandre et al., 2012), BIN analysis (Ratnasingham & Hebert, 2013), morphological identification, and evolutionary trees. Concurrently, a barcode database for the fish in the Ili River Valley was built, laying the groundwork to support the conservation of local fish resources.

## 2 MATERIALS AND METHODS

All animal handling and experimental procedures in this study adhered to the ethical guidelines and received approval from the Animal Care and Use Committee at the Institute of Hydrobiology, Chinese Academy of Sciences (Approval ID: IHB 2013724).

### 2.1 Sample collection

**FIGURE 1:**
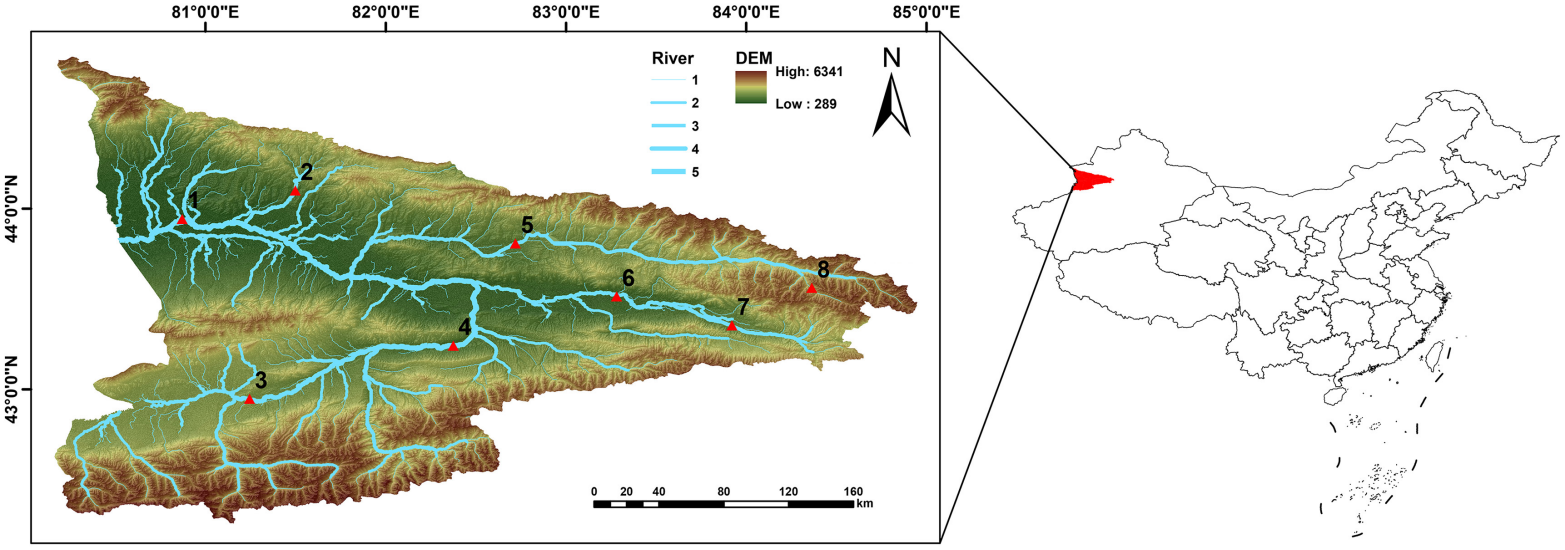
Map of sampling sites in the Ili Valley. The names of the sampling sites are as follows: 1. Xiheba village; 2. Kalayagaqi Village; 3. Moyincang Village; 4. Qiafuqihai reservoir; 5. Tastogan Village; 6. Xinhua East Road; 7. Tayiasu Village; 8. Qiaoama.

In 2023, eight sampling sites were set up in the mainstream of the Ili River and its tributaries, the Kash River, the Gongnais River, and the Tekes River, in June–July during the abundant water period and September–October during the dry water period (Figure 1). No collection or sampling permits were needed to sample at these locations from the local relevant authority. To collect fish samples, we employed various fishing tools, including dip nets, casting nets, ground cages with 3 cm mesh, and composite gill nets. The duration of each sampling session was 12 h. Fish samples collected in this study were identified using the Barbinae Cypridae of China (Wu, 1982) and Xinjiang yu lei zhi (Guo, 2012) as references, and the basic biological information of the fish samples was recorded. A moderate amount of muscle tissue or fin rays from 660 fish samples were collected and stored in liquid nitrogen for subsequent COI barcode analysis. Then fish samples were either released back into the water or preserved as specimens by soaking in the 4% formaldehyde solution.

### 2.2 DNA extraction, amplification and sequencing

We used the omega DNA extraction kit to extract DNA from muscle or fin ray samples, strictly following the kit's instructions for the procedure. The barcode region of the mitochondrial COI gene was amplified using the following fish‐specific primers: FishF1‐5′TCAACCAACCACAAAGACATTGGCAC3′; FishR1‐5′TAGACTTCTGGGTGGCCAAAGAATCA3′ (Ward et al., 2005). The total volume of the amplification reaction was 50 μL, including 25 μL 2XTaq Maxter Mix, 2 μL F‐primers, 2 μL R‐primers, 1 μL DNA, and 20 μL sterile distilled water (Yeasen Biotechnology (Shanghai) Co., Ltd.). Thermal cycling conditions of the PCR were set as follows: pre‐denaturation at 94°C for 5 min; denaturation at 94°C for 30 s, annealing at 55°C for 30 s, and extension at 72°C for 1 min for 35 cycles; final extension at 72°C for 10 min; and eventually storage at 4°C for 59 min. The PCR products were detected by 1% agarose gel electrophoresis. The products showing successful amplification were selected and sent to the Wuhan Aikangjian Biotechnology Co., Ltd. for bidirectional sequencing.

### 2.3 Data analysis

The sequences were edited and assembled using SeqMan in the DNASTAR software package (DNASTAR, Inc., Madison, WI, USA). In order to evaluate the accuracy of morphological identification, the sequence was uploaded to the NCBI database for comparison. Similar to the study of Shen et al., sequences that exhibited more than 97% similarity were classified as homologous, setting a threshold of 3% (Shen et al., 2016). Sequences were aligned by the MUSCLE algorithm (Edgar, 2004) in MEGA 11 (Tamura et al., 2021), and sequences at both ends of the DNA barcode were trimmed to get a consistent length of 576 bp for further analysis. Codon site information and base content were calculated by MEGA 11. The genetic distances both within and between species were calculated by using the Kimura 2‐parameter (K2P) model (Kimura, 1980) to assess the DNA barcode gap for all species, defined as the divergence between the maximum intra‐species distance and the minimum inter‐species distance, with the bootstrap values set to 1000.

The NJ tree was constructed based on the K2P model in MEGA 11, with the bootstrap testing was performed for 1000 times. The Gaps Data treatment was set as Partial Deletion, with the threshold set at 50%. For cluster analysis of the sequences, we utilized the Jukes‐Cantor (JC69), Kimura (K80) TS/TV and Simple Distance models from ABGD website (https://bioinfo.mnhn.fr/abi/public/abgd/). The minimum and maximum intraspecific genetic distances were set at 0.001 (P_min_) and 0.008 (P_max_), respectively, with all other parameters at their default settings. The number of haplotypes, nucleotide diversity (Pi) and haplotype diversity (Hd) were calculated to evaluate the genetic diversity of species in this region using DnaSP 6.0 (Rozas et al., 2017).

Given the inclusion of an unidentified species, the *Silurus* samples from this study were uploaded to the BOLD for BIN analysis. And nearly all available COI sequences of the genus *Silurus* from the BOLD database were downloaded to construct a NJ tree for further analysis. The parameters for the NJ tree were as previously mentioned. Subsequently, we created a haplotype network using PopART (Leigh & Bryant, 2015) to analyze the genetic structure of the undetermined species.

## 3 RESULTS

### 3.1 Sequence information

This study successfully obtained a total of 660 DNA barcode sequences, all of which were 576 bp in length. Following morphological identification and comparison with the NCBI database, we identified a total of 20 species, including an unspecified species (*Silurus* sp.), belonging to 4 orders, 6 families, and 16 genera (Table 1). After careful evaluation, *Paramisgurnus dabryanus* was eventually reclassified as *Misgurnus bipartitus*. The morphological identification results of *Silurus meridionalis* were quite different from the results of the NCBI database, so it was tentatively identified as *Silurus* sp. The 660 COI sequences had 341 highlight conserved sites, 234 highlight variable sites and 232 highlight parsimony informative sites. The sequences contained 53 transition sites and 34 transversion sites, resulting in a transitions/transversions ratio (R) of 1.55. The highest variability was observed at the third codon position. In the sequence, the G content was predominant at the first codon site; T was the most abundant base, significantly exceeding others, at the second codon position; The content of A at the third codon site was the highest. Overall, the combined content of A + T (53.86%) was greater than that of G + C (46.14%), showing the bias of A + T (Table 2). This finding aligns with the observed preference for higher AT over GC content in the base composition of the COI gene across various fish species (Habib et al., 2023; López et al., 2019; Ward et al., 2005).

**TABLE 1:** DNA barcoding similarity comparison.

Morphological identification	NCBI		
	Max score	Max ID (%)	Species name
*Gymnodiptychus dybowskii*	1267	100	*Gymnodiptychus dybowskii*
*Diptychus maculatus*	1280	100	*Diptychus maculatus*
*Cyprinus carpio*	1253	100	*Cyprinus carpio*
*Schizothorax pseudoaksaiensis*	1258	100	*Schizothorax pseudoaksaiensis*
*Carassius auratus*	1260	100	*Carassius auratus*
*Abbottina rivularis*	1254	100	*Abbottina rivularis*
*Rhodeus ocellatus*	1192	99.85	*Rhodeus ocellatus*
*Hypophthalmichthys molitrix*	1262	100	*Hypophthalmichthys molitrix*
*Hemiculter leucisculus*	1245	99.71	*Hemiculter leucisculus*
*Pseudorasbora parva*	1256	100	*Pseudorasbora parva*
***Paramisgurnus dabryanus***	**1245**	**99.27**	***Misgurnus bipartitus***
*Triplophysa labiata*	1245	99.85	*Triplophysa labiata*
*Triplophysa strauchii*	1260	100	*Triplophysa strauchii*
*Triplophysa dorsalis*	1201	99.54	*Triplophysa dorsalis*
*Triplophysa stolickai*	1192	99.69	*Triplophysa stolickai*
***Silurus meridionalis***	**1254**	**100**	***Silurus soldatovi***
	**1254**	**100**	***Silurus asotus***
*Silurus asotus*	1247	100	*Silurus asotus*
*Coregonus peled*	1240	99.85	*Coregonus peled*
*Oncorhynchus mykiss*	1253	99.85	*Oncorhynchus mykiss*
*Perca schrenkii*	1234	100	*Perca schrenkii*

*Note*: Species with divergence in morphological identification and NCBI matching identification are highlighted in bold font.

**TABLE 2:** Nucleotide bases information of DNA barcode sequence.

Nucleotide base	Total frequency (%)	1st (%)	2nd (%)	3rd (%)
T	29.29	18.43	42.16	27.23
C	28.74	26.27	30.24	29.69
A	24.57	26.25	14.06	33.40
G	17.40	29.04	13.54	9.68

### 3.2 Genetic distance

**FIGURE 2:**
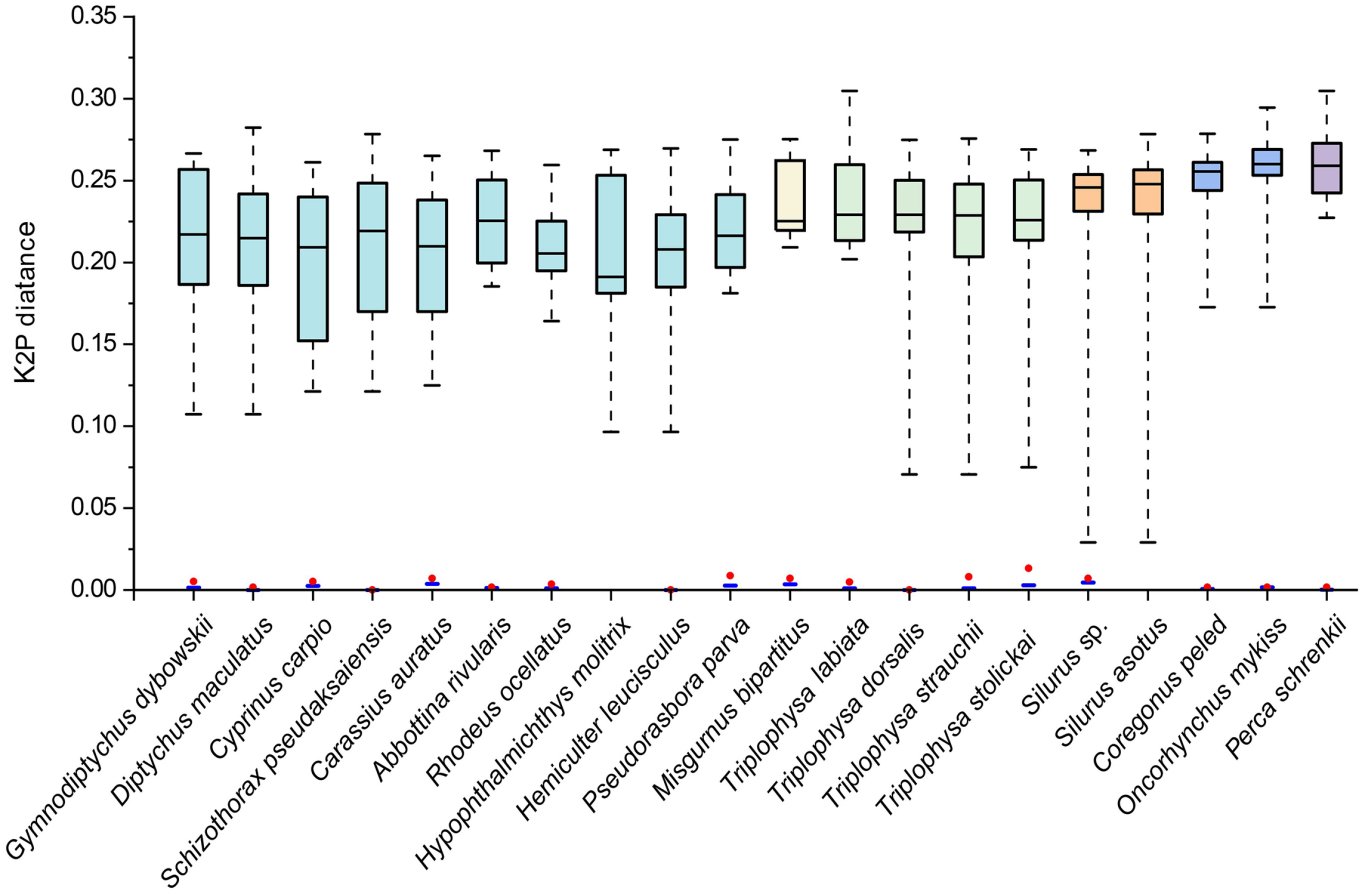
DNA barcoding gaps of interspecific and intraspecific genetic distance based on the K2P model. Each box from top to bottom represents the maximum, upper quartile, median, lower quartile and minimum of the interspecific distance. Blue line: Mean intraspecific genetic distance; Red point: Maximum intraspecific genetic distance.

Genetic distances were not calculated for *S. asotus* and *H. molitrix* (only one sample). The mean intraspecific distance for the remaining 18 species was 0%–0.47%, with the highest intraspecific distance of 0.47% observed in *Silurus* sp. The intraspecific genetic distances of *Schizothorax pseudaksaiensis*, *Hemiculter leucisculus* and *Triplophysa dorsalis* are all 0. At high taxonomic levels, the species composition is monotonous. Without considering *Silurus* sp., only the genus *Triplophysa* comprises multiple species. The range of genetic distances between these species varies from 7% to 23%. The interspecific genetic distance of *Triplophysa labiata* is as high as 20%, which is significantly greater than interspecific distance of other *Triplophysa* species, demonstrating remarkable genetic divergence. Similarly, the species composition at the family level also exhibits homogeneity, with half of the species belonging to the Cypriniformes. Except for *S. asotus* and *H. molitrix*, the maximum intraspecific genetic distance among the remaining species was smaller than the minimum interspecific distance, forming a clear barcode gap (Figure 2).

### 3.3 Species delimitation

**FIGURE 3:**
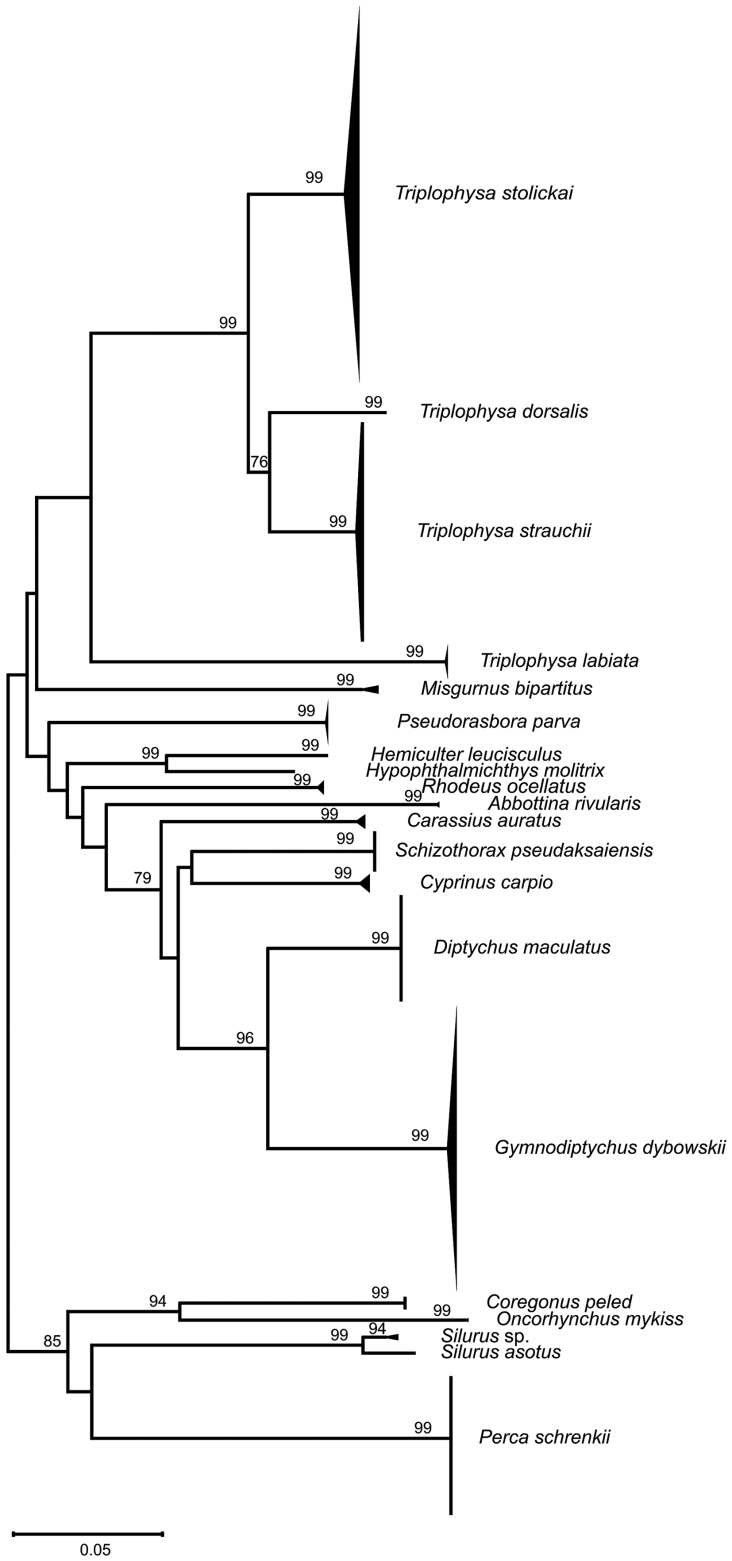
NJ tree of 660 COI barcodes based on the K2P model. Bootstrap values higher than 70 are shown.

The NJ tree based on the K2P model showed that all fish samples were defined as 20 OTUs (Figure 3). Different species were clustered into separate branches, with a support rate exceeding 93% at the species level. Cyprinidae and Cobitidae were aggregated separately and then clustered to form a monophyletic branch of the Cypriniformes. Siluriformes, Salmoniformes and Perciformes each aggregated into their respective separate branches. The topological structure of the NJ tree based on DNA barcoding was consistent with the results of morphological classification, indicating that DNA barcoding has a good ability to identify fish species in the Ili River Valley.

The study employed three genetic distance models from the ABGD website—Jukes‐Cantor (JC69), Kimura (K80) TS/TV, and Simple Distance—to identify the species (Table 3). Both the Jukes‐Cantor and Kimura (K80) TS/TV models yielded initial and recursive partitions ranging from 19 to 86 OTUs, while the initial partition and recursive partition of the Simple Distance model are always maintained at 19 OTUs. Integrating the results from these three models, ABGD ultimately classified the species into 19 OTUs. However, species were divided into 20 OTUs based on morphological characteristics and tree structure. When P was 0.0063, both the Jukes‐Cantor and Kimura (K80) TS/TV models classified the species into 20 OTUs but failed to distinguish between *Silurus* sp. and *S. asotus*. Instead, they split *Pseudorasbora parva* into 2 OTUs. The division of *P. parva* into different OTUs, with one group found in tributaries, suggests that geographical isolation may have contributed to this differentiation. Alternatively, the species within the OTU could be invasive from another region. In addition, all three models grouped *Silurus* sp. and *S. asotus* into a single OTU, indicating that *Silurus* sp. might be *S. asotus* or have a very close genetic relationship with it.

**TABLE 3:** ABGD analysis is based on three models.

Model	X	Partition	Prior intraspecific divergence									
			0.0010	0.0013	0.0016	0.0020	0.0025	0.0032	0.0040	0.0050	0.0063	0.0080
Jukes‐Cantor	1.5	Initial	86	86	86	19	19	19	19	19	19	19
		Recursive	86	86	86	22	22	22	21	21	20	19
Kimura (K80) TS/TV	1.5	Initial	86	86	86	19	19	19	19	19	19	19
		Recursive	86	86	86	22	22	22	21	21	20	19
Simple Distance	1.5	Initial	19	19	19	19	19	19	19	19	19	19
		Recursive	19	19	19	19	19	19	19	19	19	19

*Note*: X, relative gap width.

### 3.4 Analysis of an undetermined species

There are three specimens identified as *Silurus* sp. in this study, and their NCBI database comparison results significantly diverged from their morphological identifications. Consequently, nearly all available COI sequences associated with the genus *Silurus* from the BOLD database were downloaded, aiming to explain the result on a larger scale.

**FIGURE 4:**
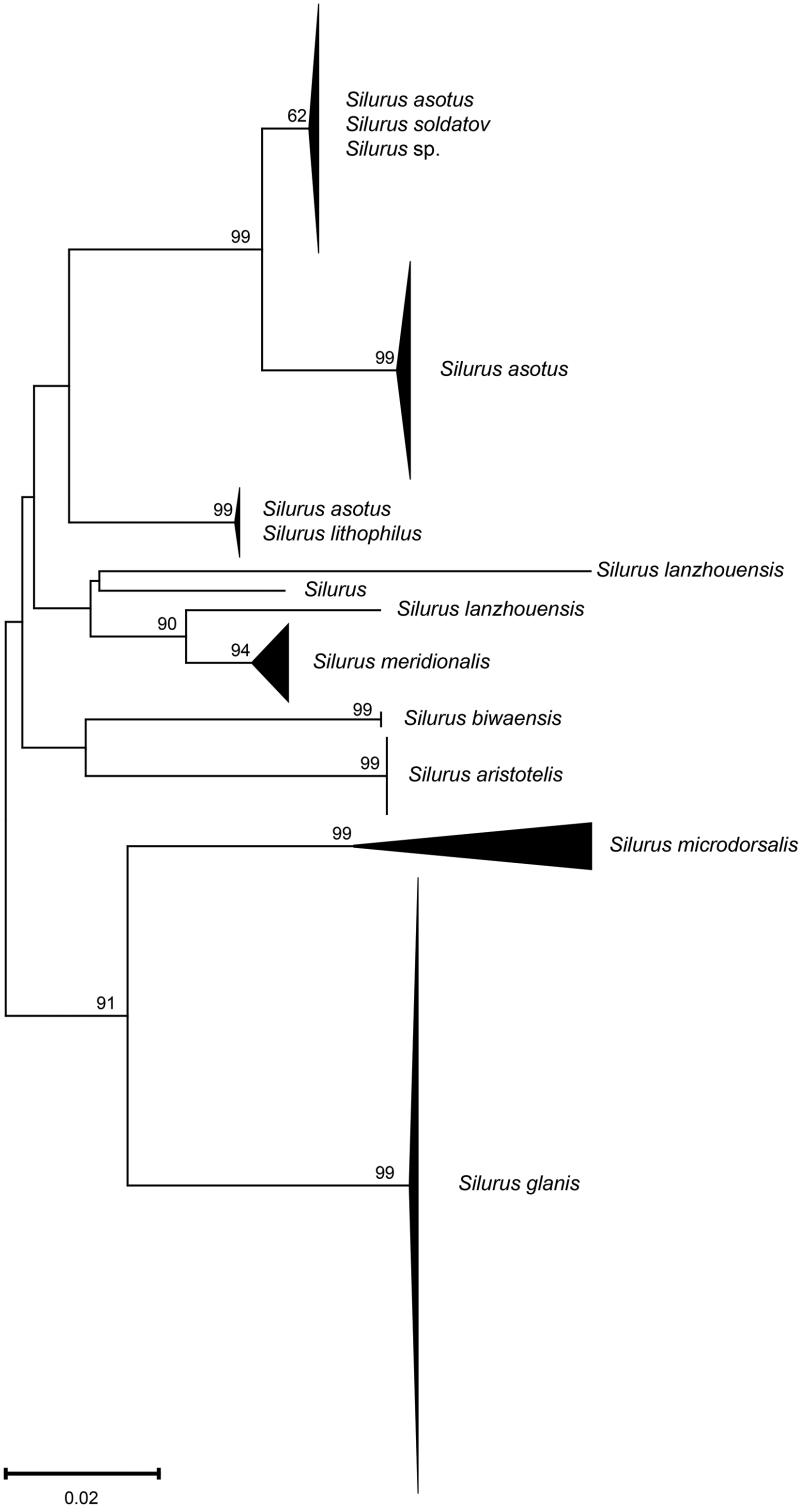
NJ tree of the genus *Silurus* based on COI sequences downloaded from BOLD and collected. *Silurus* is derived from the BOLD database. *Silurus* sp. are derived from the samples collected.

**FIGURE 5:**
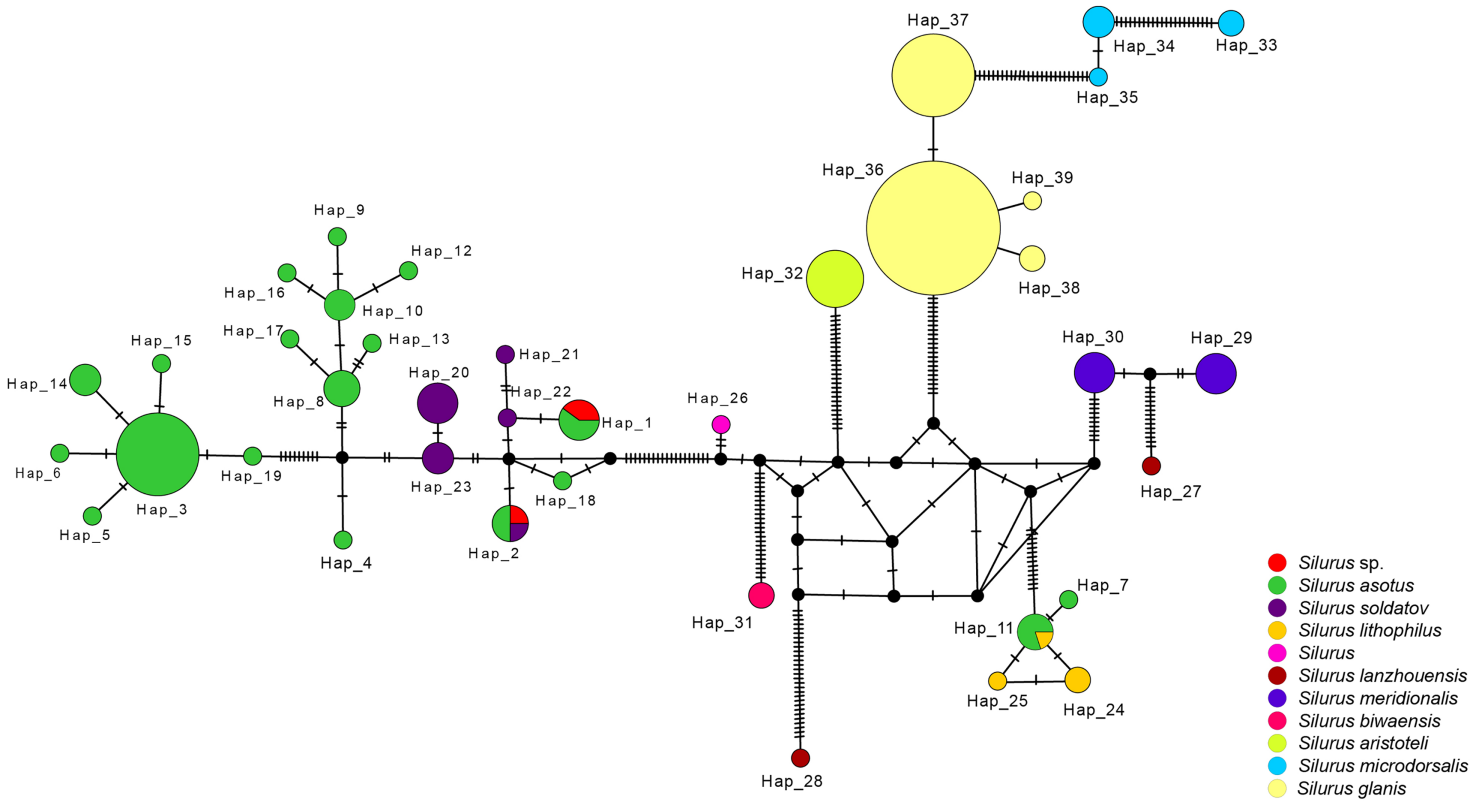
Haplotype network of the genus *Silurus*. A black circle indicates a missing intermediate step between observed haplotypes. A short line represents the mutation step. The size of the circle is proportional to the number of samples.

The *Silurus* species collected in this study were assigned to two BINs (BOLD:ACQ3910 contained *S. asotus*; BOLD:AAF8879 contained *S. asotus* and *Silurus soldatov*). The *Silurus* sp. are all classified as BOLD:AAF8879. Additionally, we observed that several BINs associated with *S. asotus* overlapped with either *S. soldatov* or *Silurus lithophilus*, indicating the existence of a disordered genetic lineage within *S. asotus*. In the NJ tree (Figure 4), *S. asotus* presents three distinct evolutionary lineages. The first branch comprises solely *S. asotus*; the second branch exhibits an intertwined structure involving both *S. asotus* and *S. lithophilus*; and the third branch includes *S. asotus*, *Silurus soldatov*, and *Silurus* sp. In addition, *S. lanzhouensis* also formed two branches. The remaining species of the *Silurus* genus formed distinctly independent branches. According to the haplotype network structure of the genus *Silurus*, the *Silurus* sp. generated two haplotypes, both of which were shared haplotypes (Figure 5). One of the haplotypes was shared with *S. soldatovi* and *S. asotus*, while the other haplotype was shared exclusively with *S. asotus*. *S. asotus*, and *S. lithophilus* also exhibited the same phenomenon of shared haplotypes. *S. lanzhouensis* formed two clearly segregating haplotypes, similar to the NJ tree structure. Furthermore, *S. meridionalis* exhibited clear divergence from the *Silurus* sp., whether in the NJ tree or haplotype network, confirming the initial morphological identification error and indicating that *Silurus* sp. is not the same as *S. meridionalis*.

### 3.5 Genetic diversity

The 660 sequences generated a total of 86 haplotypes (Table 4). *Triplophysa stolickai* exhibited the highest number of haplotypes, *Cyprinus carpio* showed the greatest haplotype diversity, and *Silurus* sp. had the highest nucleotide diversity. However, the haplotype diversity and nucleotide diversity of *Schizothorax pseudoaksaiensis* were both 0.

**TABLE 4:** DNA diversity parameters for 20 species.

Species	*N*	Nh	Hd ± SD	Pi ± SD
Gymnodiptychus dybowskii*	136	10	0.606 ± 0.034	0.00135 ± 0.00013
Diptychus maculatus*	50	2	0.040 ± 0.038	0.00007 ± 0.00007
*Cyprinus carpio*	8	4	0.821 ± 0.101	0.00255 ± 0.00062
Schizothorax pseudoaksaiensis*	19	1	0.000 ± 0.000	0.00000 ± 0.00000
*Carassius auratus*	7	3	0.667 ± 0.160	0.00381 ± 0.00082
*Abbottina rivularis*	3	2	0.667 ± 0.314	0.00116 ± 0.00055
*Rhodeus ocellatus*	7	2	0.286 ± 0.196	0.00099 ± 0.00068
*Hypophthalmichthys molitrix*	1	1	–	–
*Hemiculter leucisculus*	2	1	–	–
*Pseudorasbora parva*	22	4	0.333 ± 0.124	0.00263 ± 0.00093
*Misgurnus bipartitus*	4	2	0.500 ± 0.265	0.00348 ± 0.00184
Triplophysa labiata*	17	4	0.419 ± 0.141	0.00095 ± 0.00037
Triplophysa strauchii*	103	12	0.286 ± 0.059	0.00094 ± 0.00024
Triplophysa dorsalis*	2	1	–	–
Triplophysa stolickai*	195	28	0.550 ± 0.043	0.00281 ± 0.00031
*Silurus* sp.	3	2	0.667 ± 0.314	0.00464 ± 0.00219
*Silurus asotus*	1	1	–	–
*Coregonus peled*	6	2	0.333 ± 0.215	0.00058 ± 0.00037
*Oncorhynchus mykiss*	2	2	–	–
Perca schrenkii*	72	2	0.106 ± 0.048	0.00019 ± 0.00008

*Note*: Native species marked with *.

Abbreviations: Hd, haplotype diversity; *N*, number of samples; Nh, number of haplotypes; Pi, nucleotide diversity.

As a result of the artificial transplantation of commercially valuable fish species, only 8 of the 20 species collected were native to the Ili River Valley: *Gymnodiptychus dybowskii*, *Diptychus maculatus*, *S. pseudoaksaiensis*, *Triplophysa labiata*, *Triplophysa strauchii*, *Triplophysa dorsalis*, *T. stoliczkae* and *Perca schrenkii*. Among these, 4 of the 8 indigenous species exhibited a low number of haplotypes (<3). For *S. pseudoaksaiensis* and *T. dorsalis*, only a single haplotype was identified in each. Meanwhile, *D. maculatus* and *P. schrenkii* each presented with two distinct haplotypes. Haplotype diversity (Hd) values for native species (with ≥3 samples) varied from 0 to 0.606, while nucleotide diversity (Pi) values ranged from 0 to 0.00281. Notably, the Pi values for all species in this study fell below the critical threshold (Grant & Bowen, 1998), indicating that the overall genetic diversity of fishes in this region is rather low.

## 4 DISCUSSION

With the emergence of DNA barcoding technology, the field of biological taxonomy has seen a tremendous breakthrough (April et al., 2011). Rapid and precise species identification is a prerequisite and basis for ecological conservation (Rasmussen & Morrissey, 2009). Increasingly, research demonstrates that DNA barcoding holds great potential in fish classification, the discovery of cryptic species, and biodiversity conservation, surpassing the capabilities of traditional taxonomy, which often relies on subjective expertise (Chen et al., 2015; Hebert et al., 2004; Li et al., 2010). In this study, we collected a total of 660 fish samples. All sequences do not contain deletions, insertions or stop codons, proving that they are high‐quality. Similar to the fishes in the Taiwan Strait (Bingpeng et al., 2018), there is a high frequency of transitions and transversions at the third codon position. The base contents of these sequences are A (24.57%), T (29.29%), G (17.40%), and C (28.74%), respectively. This composition indicates a higher AT content compared to GC, showing a clear bias, which is related to selection pressure (Bingpeng et al., 2018). By comparison on the NCBI website, 20 species are identified (including *Silurus* sp.), all of which showed over 98% sequence similarity. It is noteworthy that a species initially identified as *P. dabryanus* through morphological analysis was correctly identified as *M. bipartitus* after comparison with the NCBI database. This correction highlights the significant advantage of DNA barcoding in distinguishing species with similar morphological characteristics.

For most species, intraspecific genetic distances based on the K2P model are usually less than 2% (Hebert, Ratnasingham, & deWaard, 2003). The mean intraspecific genetic distance of fish in the Ili River Valley ranged from 0 to 0.47%, which is significantly lower than the threshold range of 2%. In addition, Hebert et al. also proposed the “10×” rule, which means that genetic variation between species is more than 10 times greater than within species (Hebert et al., 2004). And, the larger the genetic gap both between and within species, the more effective DNA barcoding is for identification. In this study, aside from two species represented by only one sample, the maximum genetic distances within species are significantly smaller than the minimum genetic distances between species, showing obvious DNA barcode gaps. The divergence in genetic distances within and between species are also in accordance with the 10‐fold criterion. In the NJ tree, 20 species can be clearly separated with high bootstrap values. Families such as Cyprinidae, Cobitidae, Nemacheilidae, Siluridae, Salmonidae, and Percidae each form their own monophyletic branches. Unlike the cases with marine fishes in Guangdong Province (Li, 2022) and fishes in the Three Gorges Reservoir Area (He, 2023), there are no situations where species could not be accurately clustered at the genus level. Although there is a case where a species is difficult to identify, DNA barcoding based on the COI gene had a 95% success rate in this study. This rate surpasses the identification success rates reported for North American freshwater fishes (90%) (April et al., 2011) and Maranhão fishes (92.19%) (Nascimento et al., 2016), underscoring the substantial applicability of DNA barcoding. In conclusion, the DNA barcoding based on the COI gene shows considerable promise in the Ili River valley, which can provide a rapid and accurate identification method for the investigation, conservation and sustainable management of local fish resources.

In the traditional DNA barcoding method, the threshold for species delimitation is set to a constant value. However, the genetic thresholds of different species have certain divergence due to factors such as the species formation time, population size and geographical distribution (Collins & Cruickshank, 2013; Zhang et al., 2022). ABGD analysis automatically detects “barcode gaps” in the sequence based on a priori intraspecific divergence, thus reducing the uncertainty of threshold setting to some extent (Puillandre et al., 2012). Species clustering, determined via the ABGD website, aligned most closely with the phylogenetic tree and morphological identification outcomes when *P* ranged between 0.63% and 0.8%. Based on this, we believe that setting the DNA barcoding threshold value in the Ili River Valley to between 0.6% and 0.9% is the most suitable.

The mitochondrial COI gene is commonly utilized as a marker gene for DNA barcoding due to its moderate rate of evolution and adequate sequence variation (Pentinsaari et al., 2016; Tadmor‐Levi et al., 2023). However, since the COI gene is maternally inherited, interspecies hybridization or endosymbiont infection can impact the accuracy of DNA barcoding, revealing some limitations of relying solely on the COI gene (Wu et al., 2011). According to the species delineation of *Silurus* sp., ABGD analyses differ from morphological identifications and BIN analyses, with ABGD grouping *Silurus* sp. with *S. asotus* into one OTU. With the exception of *S. asotus* and *Silurus* sp. in the study, *S. asotus* is also intermixed with *S. soldatov* and *S. lithophilus* in the phylogenetic tree and forms shared haplotypes with them, suggesting a close genetic relationship among them. Consequently, we speculate that *S. asotus* may have a complex evolutionary history, such as hybridization, gene introgression, historical population structure change, or rapid radiative evolution, which has resulted in an unusually close and ambiguous relationship with other species within the genus *Silurus*. Simultaneously, the COI gene, due to its limited sequence length and relatively low amount of genetic information, as well as its moderate evolutionary rate, may present blind spots when identifying closely related species. A recent detailed study demonstrated that ITS sequences, which evolve more rapidly, offer greater accuracy for identifying phylogenetic species compared to the COI gene, which has a moderate rate of evolution (Mao, 2023). *S. lanzhouensis* also exhibits two distinct clades, but considering that there are only two samples of *S. lanzhouensis* in the BOLD database, these two samples may show significant genetic differences due to random genetic drift or geographical isolation. In summary, the taxonomic status of the genus *Silurus* is currently complex and unclear, necessitating further in‐depth morphological and genetic research.

Regarding the identification of *Silurus* sp., this study presents two hypotheses. The first speculation is that *Silurus* sp. may be the hybridization progeny of *S. soldatovi* and *S. asotus*, displaying characteristics that are intermediate to those of its parents. A study has also found that *S. soldatovi* and *S. asotus* have a close kinship and can be hybridized with each other (Hu et al., 2005). However, *S. soldatovi* is naturally distributed in the middle and lower reaches of the Heilongjiang and Liao Rivers in China (Liu et al., 2013), with no historical records of its presence in the Ili Valley. Based on this speculation, we conclude that the unidentified species may also be *S. asotus*. The genetic distance between the *Silurus* sp. and the *S. asotus* is 2.92%, which is above the 2% threshold criterion for species differentiation. We think that this *Silurus* sp. could be a recently diverged cryptic species of *S. asotus*, likely a result of incomplete genealogical sorting between the two species (Heled & Drummond, 2010). This phenomenon, where different segments of the genome have different rates of evolution and conservation, results in the construction of phylogenetic trees based on COI genes that do not reflect the true evolutionary relationships of the species. Similar situations have been found in *Triplophysa* (Wang et al., 2020), the Qinghai‐Tibet plateau endemic species (Shen et al., 2019) and *Laemolyta* (Ramirez & Galetti, 2015). We will collect more samples and combine several molecular markers to explore the clustering disorders caused by interspecific hybridization, gene introgression, and recent species differentiation.

Anthropogenic disturbances and landscape fragmentation can cause fish populations to decline, which in turn diminishes species diversity. Among the native fishes with more than three samples, five species exhibited both low haplotype and nucleotide diversity, indicating that these species may have experienced a bottleneck effect recently. Only two species showed high haplotype diversity alongside low nucleotide diversity, suggesting they experienced a bottleneck effect followed by rapid expansion, resulting in the accumulation of a large amount of variation within the population (Grant & Bowen, 1998; He, 2023). In addition, exotic economic fish species also showed a low level of nucleotide diversity. Haplotype diversity and nucleotide diversity are important indicators of genetic diversity (Féral, 2002). The data show that the level of genetic diversity among indigenous species is extremely low. Apart from this, the native fish, *Schizothorax argentatus* has not been found in recent years (Liu et al., 2022). The above evidence points to a gradual decrease in native fish populations. Fish resources play a vital role in ecological construction. In order to prevent the further decline of fish diversity in the Ili River Valley, we should strengthen the protection and research of wild fish resources in the area.

In this study, DNA barcoding was applied to identify fish species for the first time in the Ili River Valley of Xinjiang, China, covering more than half of the fishes in the region. Our findings reveal that the area faces significant ecological risk and is on a declining ecological trend. Furthermore, the accuracy of our DNA barcode database has been confirmed, which will provide an important scientific foundation for the subsequent ecological assessment and biodiversity conservation in the Ili River Valley.

## AUTHOR CONTRIBUTIONS

**Ling‐Ling Zheng:** Conceptualization (equal); data curation (equal); formal analysis (lead); funding acquisition (supporting); investigation (equal); methodology (lead); project administration (supporting); resources (supporting); software (lead); supervision (supporting); validation (equal); visualization (equal); writing – original draft (lead); writing – review and editing (lead). **Dan Yu:** Conceptualization (supporting); data curation (equal); formal analysis (supporting); funding acquisition (supporting); investigation (equal); methodology (equal); project administration (supporting); resources (equal); software (equal); supervision (supporting); validation (equal); visualization (equal); writing – original draft (supporting); writing – review and editing (equal). **Ning Sun:** Conceptualization (equal); data curation (supporting); formal analysis (supporting); funding acquisition (supporting); investigation (supporting); methodology (equal); project administration (supporting); resources (supporting); software (supporting); supervision (supporting); validation (supporting); visualization (supporting); writing – original draft (supporting); writing – review and editing (supporting). **Cheng Wang:** Conceptualization (supporting); data curation (supporting); formal analysis (supporting); funding acquisition (supporting); investigation (supporting); methodology (supporting); project administration (supporting); resources (supporting); software (supporting); supervision (supporting); validation (supporting); visualization (supporting); writing – original draft (supporting); writing – review and editing (supporting). **Wen‐Jun Chen:** Conceptualization (supporting); data curation (supporting); formal analysis (supporting); funding acquisition (supporting); investigation (supporting); methodology (supporting); project administration (supporting); resources (supporting); software (supporting); supervision (supporting); validation (supporting); visualization (supporting); writing – original draft (supporting); writing – review and editing (supporting). **Zu‐Fa Ding:** Conceptualization (supporting); data curation (supporting); formal analysis (supporting); funding acquisition (supporting); investigation (supporting); methodology (lead); project administration (supporting); resources (supporting); software (supporting); supervision (supporting); validation (supporting); visualization (supporting); writing – original draft (supporting); writing – review and editing (supporting). **Shun‐Ping He:** Conceptualization (equal); data curation (equal); formal analysis (equal); funding acquisition (supporting); investigation (supporting); methodology (supporting); project administration (equal); resources (equal); software (equal); supervision (equal); validation (equal); visualization (equal); writing – original draft (supporting); writing – review and editing (supporting). **Lian‐Dong Yang:** Conceptualization (lead); data curation (lead); formal analysis (equal); funding acquisition (lead); investigation (lead); methodology (equal); project administration (lead); resources (lead); software (equal); supervision (lead); validation (lead); visualization (lead); writing – original draft (equal); writing – review and editing (equal).

## FUNDING INFORMATION

This research was supported by the Third Xinjiang Scientific Expedition Program (Grant No.2021xjkk0500) and the National Natural Science Foundation of China (32170480).

## CONFLICT OF INTEREST STATEMENT

The authors declare there are no competing interests.

## Data Availability

All new DNA sequences (Accession numbers: SIL001‐24‐SIL004‐24, ILI001‐24‐ILI656‐24) used in this study were uploaded to Bold Systems (www.boldsystems.org).
